# (±)-Ethyl 6-(6-meth­oxy-2-naphth­yl)-4-(4-methyl­phen­yl)-2-oxocyclo­hex-3-ene-1-carboxyl­ate

**DOI:** 10.1107/S1600536809016341

**Published:** 2009-05-07

**Authors:** Hongqi Li, A. N. Mayekar, B. Narayana, H. S. Yathirajan, William T. A. Harrison

**Affiliations:** a Key Laboratory of Science & Technology of Eco-Textiles, Ministry of Education, College of Chemistry, Chemical Engineering & Biotechnology, Donghua University, Shanghai 201620, People’s Republic of China; b Department of Studies in Chemistry, University of Mysore, Manasagangotri, Mysore 570 006, India; c Department of Studies in Chemistry, Mangalore University, Mangalagangotri 574 199, India; d Department of Chemistry, University of Aberdeen, Aberdeen AB24 3UE, Scotland

## Abstract

In the title compound, C_27_H_26_O_4_, the dihedral angle between the naphthalene ring system and the benzene ring is 73.10 (5)°. In the crystal, a weak C—H⋯O inter­action occurs. Two C—H groups of the cyclo­hexene ring are disordered over two sets of sites in a 0.796 (5):0.204 (5) ratio, which corresponds to partial overlap of the two enanti­omeric mol­ecules.

## Related literature

For related structures, see: Fischer *et al.* (2007*a* ▶,*b* ▶, 2008 ▶).
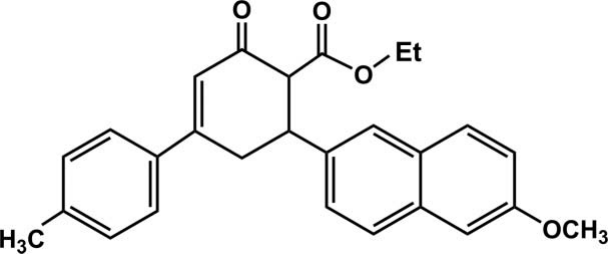


## Experimental

### 

#### Crystal data

C_27_H_26_O_4_
*M* *_r_* = 414.48
Monoclinic, 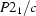

*a* = 18.8013 (10) Å
*b* = 11.3604 (6) Å
*c* = 10.2356 (6) Å
β = 93.430 (2)°
*V* = 2182.3 (2) Å^3^
*Z* = 4
Mo *K*α radiation
μ = 0.08 mm^−1^
*T* = 296 K
0.44 × 0.38 × 0.22 mm

#### Data collection

Bruker SMART CCD diffractometer
Absorption correction: none
26105 measured reflections
4269 independent reflections
3385 reflections with *I* > 2σ(*I*)
*R*_int_ = 0.024

#### Refinement

*R*[*F* ^2^ > 2σ(*F* ^2^)] = 0.050
*wR*(*F* ^2^) = 0.147
*S* = 1.08
4269 reflections
292 parameters
H-atom parameters constrained
Δρ_max_ = 0.28 e Å^−3^
Δρ_min_ = −0.23 e Å^−3^

### 

Data collection: *SMART* (Bruker, 2006 ▶); cell refinement: *SAINT* (Bruker, 2006 ▶); data reduction: *SAINT*; program(s) used to solve structure: *SHELXS97* (Sheldrick, 2008 ▶); program(s) used to refine structure: *SHELXL97* (Sheldrick, 2008 ▶); molecular graphics: *ORTEP-3* (Farrugia, 1997 ▶); software used to prepare material for publication: *SHELXL97*.

## Supplementary Material

Crystal structure: contains datablocks global, I. DOI: 10.1107/S1600536809016341/dn2449sup1.cif

Structure factors: contains datablocks I. DOI: 10.1107/S1600536809016341/dn2449Isup2.hkl

Additional supplementary materials:  crystallographic information; 3D view; checkCIF report

## Figures and Tables

**Table 1:** Hydrogen-bond geometry (Å, °)

*D*—H⋯*A*	*D*—H	H⋯*A*	*D*⋯*A*	*D*—H⋯*A*
C26—H26*A*⋯O3^i^	0.97	2.42	3.332 (4)	157

Symmetry code: (i) 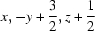.

**Fig. 1.**
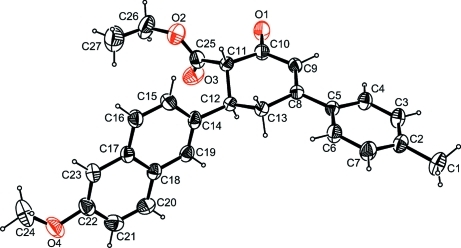
A view of (I) with displacement ellipsoids for the non-hydrogen atoms drawn at the 30% probability level. Only the major disorder component is shown.

## supplementary crystallographic information

### Comment

The crystal structures of (8RS,9SR)-ethyl 4-(3-bromothien-2-yl)- 6-(2-furyl)-2-oxocyclohex-3-ene-1-carboxylate, (7RS,8SR)-ethyl 6-(1,3-benzodioxol-5-yl)-3-(3-bromo-2-thienyl)-2-oxocyclohex-3- ene-1-carboxylate and ethyl 4-(3-bromo-2-thienyl)-2-oxo-6- phenylcyclohex-3-ene-1-carboxylate have been reported (Fischer *et al.*, 2007*a*,*b*, 2008). As part of our ongoing studies of cyclohexene carboxylates, we now describe the crystal structure of the title compound, (I) (Fig. 1).

The dihedral angle between the mean planes of the naphthalene (C14–C23) and benzene (C2–C7) rings is 73.10 (5)°. Atom C24 is almost coplanar with its attached benzene ring [displacement = 0.029 (3) Å]. The cyclohexene ring shows a distorted half-chair conformation, with atoms C8, C9, C10 and C13 almost coplanar (r.m.s. deviation = 0.009 Å) and C11 and C12 displaced by 0.209 (4) and -0.549 (4) Å, respectively. C11 and C12 are disordered over two sites in a 0.796 (5):0.204 (5) ratio and the minor disorder components show displacements from C8/C9/C10/C13 in the opposite sense: C11A = -0.72 (1) Å; C12A = 0.21 (1)%A. C11 and C12 are stereogenic centres: in the major disorder component, they have S and *R* conformations respectively. In the minor component, C11A and C12A have *R* and S conformations, respectively, thus the disorder corresponds to partial overlap of enantiomeric molecules. However, a fully racemic mixture is generated by crystal symmetry.

In the crystal, a weak C—H···O interaction occurs (Table 1), leading to chains of molecules propagating in [001]. There are no aromatic π···π stacking interactions in the title compound, the shortest intermolecular aromatic ring centroid–centroid separation being greater than 5.3 Å.

### Experimental

Ethyl acetoacetate (0.65 g, 5 mmol) and (2E)-3-(6-methoxynaphthalen-2-yl)-1-(4-methylphenyl)prop-2- en-1-one (1.51 g, 5 mmol) were refluxed for 6 hr in 10–15 ml of ethanol in the presence of 0.8 ml of 10% NaOH. The reaction mixture was cooled to room temperature and the reaction mass was filtered and recrystallized using acetonitrile to yield colourless blocks of (I) (m.p. 431–433 K). Analysis: found (calculated): C%, 78.15 (78.24); H%, 6.27 (6.32).

### Refinement

Atoms C11 and C12 and their attached H atoms are disordered over two sets of sites in a 0.796 (5):0.204 (5) ratio. All H atoms were placed in idealized locations (C—H = 0.93–0.98 Å) and refined as riding with *U*_iso_(H) = 1.2*U*_eq_(C) or 1.5*U*_eq_(methyl C). The methyl groups were allowed to rotate, but not to tip, to best fit the electron density.

### Crystal data

**Table:**

C_27_H_26_O_4_	*F*(000) = 880
*M**_r_* = 414.48	*D*_x_ = 1.262 Mg m^−^^3^
Monoclinic, *P*2_1_/*c*	Melting point = 431–433 K
Hall symbol: -P 2ybc	Mo *K*α radiation, λ = 0.71073 Å
*a* = 18.8013 (10) Å	Cell parameters from 9970 reflections
*b* = 11.3604 (6) Å	θ = 2.3–28.1°
*c* = 10.2356 (6) Å	µ = 0.08 mm^−^^1^
β = 93.430 (2)°	*T* = 296 K
*V* = 2182.3 (2) Å^3^	Block, colorless
*Z* = 4	0.44 × 0.38 × 0.22 mm

### Data collection

**Table:**

Bruker SMART CCD diffractometer	3385 reflections with *I* > 2σ(*I*)
Radiation source: fine-focus sealed tube	*R*_int_ = 0.024
graphite	θ_max_ = 26.0°, θ_min_ = 2.1°
ω scans	*h* = −22→23
26105 measured reflections	*k* = −14→13
4269 independent reflections	*l* = −12→12

### Refinement

**Table:**

Refinement on *F*^2^	Primary atom site location: structure-invariant direct methods
Least-squares matrix: full	Secondary atom site location: difference Fourier map
*R*[*F*^2^ > 2σ(*F*^2^)] = 0.050	Hydrogen site location: inferred from neighbouring sites
*wR*(*F*^2^) = 0.147	H-atom parameters constrained
*S* = 1.08	*w* = 1/[σ^2^(*F*_o_^2^) + (0.0575*P*)^2^ + 0.796*P*] where *P* = (*F*_o_^2^ + 2*F*_c_^2^)/3
4269 reflections	(Δ/σ)_max_ < 0.001
292 parameters	Δρ_max_ = 0.28 e Å^−^^3^
0 restraints	Δρ_min_ = −0.23 e Å^−^^3^

### Special details

**Table:**

Geometry. All e.s.d.'s (except the e.s.d. in the dihedral angle between two l.s. planes) are estimated using the full covariance matrix. The cell e.s.d.'s are taken into account individually in the estimation of e.s.d.'s in distances, angles and torsion angles; correlations between e.s.d.'s in cell parameters are only used when they are defined by crystal symmetry. An approximate (isotropic) treatment of cell e.s.d.'s is used for estimating e.s.d.'s involving l.s. planes.
Refinement. Refinement of *F*^2^ against ALL reflections. The weighted *R*-factor *wR* and goodness of fit *S* are based on *F*^2^, conventional *R*-factors *R* are based on *F*, with *F* set to zero for negative *F*^2^. The threshold expression of *F*^2^ > σ(*F*^2^) is used only for calculating *R*-factors(gt) *etc*. and is not relevant to the choice of reflections for refinement. *R*-factors based on *F*^2^ are statistically about twice as large as those based on *F*, and *R*- factors based on ALL data will be even larger.

### Fractional atomic coordinates and isotropic or equivalent isotropic displacement parameters (Å^2^)

**Table:**

	*x*	*y*	*z*	*U*_iso_*/*U*_eq_	Occ. (<1)
C1	0.69533 (14)	0.5752 (3)	0.0533 (3)	0.0902 (8)	
H1A	0.7017	0.4946	0.0284	0.135*	
H1B	0.7384	0.6038	0.0974	0.135*	
H1C	0.6843	0.6218	−0.0234	0.135*	
C2	0.63501 (10)	0.58356 (19)	0.1438 (2)	0.0620 (5)	
C3	0.62922 (11)	0.67605 (18)	0.2302 (2)	0.0653 (5)	
H3A	0.6637	0.7348	0.2338	0.078*	
C4	0.57306 (10)	0.68315 (16)	0.3114 (2)	0.0573 (5)	
H4A	0.5708	0.7463	0.3689	0.069*	
C5	0.51993 (8)	0.59785 (14)	0.30881 (16)	0.0450 (4)	
C6	0.52728 (11)	0.50508 (18)	0.2236 (2)	0.0637 (5)	
H6A	0.4934	0.4453	0.2203	0.076*	
C7	0.58344 (11)	0.4987 (2)	0.1430 (2)	0.0721 (6)	
H7A	0.5863	0.4349	0.0866	0.087*	
C8	0.45731 (8)	0.60752 (14)	0.38914 (15)	0.0430 (4)	
C9	0.45342 (9)	0.68570 (16)	0.48623 (17)	0.0514 (4)	
H9A	0.4933	0.7316	0.5081	0.062*	
C10	0.39042 (10)	0.70302 (18)	0.55941 (19)	0.0612 (5)	
C11	0.33058 (11)	0.6119 (2)	0.5394 (2)	0.0475 (7)	0.796 (5)
H11A	0.3420	0.5425	0.5935	0.057*	0.796 (5)
C12	0.32392 (11)	0.5796 (2)	0.3939 (2)	0.0434 (6)	0.796 (5)
H12A	0.3154	0.6520	0.3433	0.052*	0.796 (5)
C11A	0.3197 (4)	0.6664 (8)	0.4725 (9)	0.044 (2)*	0.204 (5)
H11B	0.3085	0.7140	0.3940	0.053*	0.204 (5)
C12A	0.3363 (4)	0.5376 (9)	0.4490 (10)	0.042 (2)*	0.204 (5)
H12B	0.3454	0.4923	0.5298	0.050*	0.204 (5)
C13	0.39476 (8)	0.52783 (14)	0.35494 (16)	0.0454 (4)	
H13A	0.4034	0.4519	0.3962	0.054*	
H13B	0.3909	0.5157	0.2610	0.054*	
C14	0.26209 (9)	0.49678 (16)	0.3617 (2)	0.0568 (5)	
C15	0.24061 (10)	0.40353 (19)	0.4418 (2)	0.0645 (5)	
H15A	0.2664	0.3881	0.5203	0.077*	
C16	0.18260 (10)	0.33559 (18)	0.4062 (2)	0.0636 (5)	
H16A	0.1693	0.2751	0.4610	0.076*	
C17	0.14295 (9)	0.35618 (16)	0.28796 (18)	0.0523 (4)	
C18	0.16472 (9)	0.44890 (16)	0.20758 (18)	0.0527 (4)	
C19	0.22422 (10)	0.51671 (16)	0.24743 (19)	0.0562 (5)	
H19A	0.2381	0.5774	0.1936	0.067*	
C20	0.12407 (12)	0.47106 (19)	0.0890 (2)	0.0675 (5)	
H20A	0.1372	0.5322	0.0351	0.081*	
C21	0.06648 (12)	0.4046 (2)	0.0531 (2)	0.0719 (6)	
H21A	0.0409	0.4200	−0.0256	0.086*	
C22	0.04508 (10)	0.3132 (2)	0.1328 (2)	0.0674 (5)	
C23	0.08194 (10)	0.28904 (19)	0.2477 (2)	0.0640 (5)	
H23A	0.0671	0.2281	0.3004	0.077*	
C24	−0.03910 (16)	0.1578 (3)	0.1573 (4)	0.1188 (11)	
H24A	−0.0841	0.1311	0.1191	0.178*	
H24B	−0.0446	0.1818	0.2461	0.178*	
H24C	−0.0050	0.0950	0.1560	0.178*	
C25	0.26128 (11)	0.6665 (2)	0.5797 (2)	0.0677 (6)	
C26	0.17635 (17)	0.6450 (3)	0.7353 (3)	0.1119 (11)	
H26A	0.1831	0.6592	0.8287	0.134*	
H26B	0.1590	0.7172	0.6939	0.134*	
C27	0.1251 (2)	0.5530 (4)	0.7114 (4)	0.1364 (13)	
H27A	0.0832	0.5701	0.7569	0.205*	
H27B	0.1451	0.4796	0.7420	0.205*	
H27C	0.1128	0.5478	0.6192	0.205*	
O1	0.38635 (9)	0.78077 (15)	0.64006 (17)	0.0873 (5)	
O2	0.24308 (9)	0.61212 (16)	0.68399 (18)	0.0907 (5)	
O3	0.22736 (11)	0.74372 (19)	0.52718 (18)	0.0983 (6)	
O4	−0.01487 (9)	0.25493 (19)	0.08413 (19)	0.0963 (6)	

### Atomic displacement parameters (Å^2^)

**Table:**

	*U*^11^	*U*^22^	*U*^33^	*U*^12^	*U*^13^	*U*^23^
C1	0.0763 (15)	0.0981 (18)	0.1006 (18)	0.0025 (14)	0.0432 (14)	0.0005 (15)
C2	0.0528 (11)	0.0674 (12)	0.0676 (12)	0.0075 (9)	0.0172 (9)	0.0070 (10)
C3	0.0563 (11)	0.0581 (11)	0.0832 (14)	−0.0086 (9)	0.0193 (10)	0.0062 (10)
C4	0.0547 (10)	0.0488 (10)	0.0697 (12)	−0.0025 (8)	0.0142 (9)	−0.0016 (8)
C5	0.0416 (8)	0.0443 (9)	0.0491 (9)	0.0030 (7)	0.0030 (7)	0.0044 (7)
C6	0.0534 (11)	0.0620 (12)	0.0772 (13)	−0.0077 (9)	0.0171 (9)	−0.0161 (10)
C7	0.0635 (12)	0.0746 (13)	0.0805 (14)	−0.0014 (10)	0.0222 (10)	−0.0232 (11)
C8	0.0427 (8)	0.0407 (8)	0.0456 (8)	0.0017 (6)	0.0019 (6)	0.0039 (6)
C9	0.0461 (9)	0.0540 (10)	0.0542 (9)	−0.0070 (8)	0.0033 (7)	−0.0056 (8)
C10	0.0559 (11)	0.0678 (12)	0.0608 (11)	−0.0095 (9)	0.0105 (9)	−0.0208 (9)
C11	0.0485 (12)	0.0476 (12)	0.0471 (13)	−0.0005 (9)	0.0084 (9)	−0.0013 (10)
C12	0.0438 (11)	0.0421 (12)	0.0446 (12)	−0.0001 (9)	0.0059 (9)	0.0000 (10)
C13	0.0474 (9)	0.0413 (8)	0.0479 (9)	−0.0007 (7)	0.0072 (7)	−0.0018 (7)
C14	0.0467 (10)	0.0538 (10)	0.0722 (12)	−0.0077 (8)	0.0218 (9)	−0.0181 (9)
C15	0.0534 (11)	0.0736 (13)	0.0653 (12)	−0.0040 (9)	−0.0049 (9)	−0.0039 (10)
C16	0.0571 (11)	0.0631 (12)	0.0705 (12)	−0.0108 (9)	0.0026 (9)	0.0089 (10)
C17	0.0427 (9)	0.0522 (10)	0.0623 (10)	−0.0002 (7)	0.0072 (8)	−0.0031 (8)
C18	0.0503 (10)	0.0481 (9)	0.0610 (10)	0.0037 (8)	0.0158 (8)	−0.0069 (8)
C19	0.0589 (11)	0.0512 (10)	0.0606 (11)	−0.0048 (8)	0.0199 (9)	−0.0086 (8)
C20	0.0771 (14)	0.0650 (12)	0.0609 (11)	0.0125 (11)	0.0102 (10)	0.0024 (10)
C21	0.0656 (13)	0.0836 (15)	0.0655 (12)	0.0183 (11)	−0.0045 (10)	−0.0097 (11)
C22	0.0470 (10)	0.0776 (14)	0.0770 (13)	0.0034 (10)	−0.0006 (9)	−0.0139 (11)
C23	0.0493 (10)	0.0646 (12)	0.0784 (13)	−0.0075 (9)	0.0050 (9)	0.0018 (10)
C24	0.0733 (17)	0.113 (2)	0.168 (3)	−0.0341 (17)	−0.0115 (19)	−0.005 (2)
C25	0.0553 (12)	0.0699 (13)	0.0790 (14)	−0.0043 (10)	0.0145 (10)	−0.0293 (12)
C26	0.103 (2)	0.119 (2)	0.120 (2)	−0.0216 (19)	0.0603 (19)	−0.0187 (19)
C27	0.113 (3)	0.143 (3)	0.156 (3)	−0.022 (2)	0.040 (2)	−0.020 (3)
O1	0.0792 (10)	0.0959 (12)	0.0898 (11)	−0.0252 (9)	0.0284 (8)	−0.0513 (9)
O2	0.0829 (11)	0.0923 (12)	0.0993 (12)	0.0043 (9)	0.0245 (10)	0.0012 (10)
O3	0.1126 (14)	0.0993 (13)	0.0851 (12)	0.0236 (12)	0.0247 (10)	−0.0004 (10)
O4	0.0593 (9)	0.1194 (15)	0.1076 (13)	−0.0101 (10)	−0.0173 (9)	−0.0175 (12)

### Geometric parameters (Å, °)

**Table:**

C1—C2	1.510 (3)	C13—H13A	0.9700
C1—H1A	0.9600	C13—H13B	0.9700
C1—H1B	0.9600	C14—C19	1.351 (3)
C1—H1C	0.9600	C14—C15	1.413 (3)
C2—C7	1.367 (3)	C15—C16	1.368 (3)
C2—C3	1.381 (3)	C15—H15A	0.9300
C3—C4	1.385 (3)	C16—C17	1.403 (3)
C3—H3A	0.9300	C16—H16A	0.9300
C4—C5	1.391 (2)	C17—C18	1.412 (3)
C4—H4A	0.9300	C17—C23	1.418 (3)
C5—C6	1.380 (3)	C18—C19	1.399 (3)
C5—C8	1.480 (2)	C18—C20	1.417 (3)
C6—C7	1.380 (3)	C19—H19A	0.9300
C6—H6A	0.9300	C20—C21	1.353 (3)
C7—H7A	0.9300	C20—H20A	0.9300
C8—C9	1.338 (2)	C21—C22	1.395 (3)
C8—C13	1.509 (2)	C21—H21A	0.9300
C9—C10	1.452 (3)	C22—C23	1.357 (3)
C9—H9A	0.9300	C22—O4	1.374 (3)
C10—O1	1.214 (2)	C23—H23A	0.9300
C10—C11	1.533 (3)	C24—O4	1.424 (4)
C10—C11A	1.610 (8)	C24—H24A	0.9600
C11—C25	1.522 (3)	C24—H24B	0.9600
C11—C12	1.531 (3)	C24—H24C	0.9600
C11—H11A	0.9800	C25—O3	1.193 (3)
C12—C14	1.517 (3)	C25—O2	1.297 (3)
C12—C13	1.531 (3)	C26—C27	1.432 (4)
C12—H12A	0.9800	C26—O2	1.438 (3)
C11A—C12A	1.519 (13)	C26—H26A	0.9700
C11A—C25	1.599 (8)	C26—H26B	0.9700
C11A—H11B	0.9800	C27—H27A	0.9600
C12A—C13	1.508 (9)	C27—H27B	0.9600
C12A—C14	1.677 (9)	C27—H27C	0.9600
C12A—H12B	0.9800		
			
C2—C1—H1A	109.5	C11A—C12A—H12B	113.6
C2—C1—H1B	109.5	C14—C12A—H12B	113.7
H1A—C1—H1B	109.5	H11A—C12A—H12B	34.6
C2—C1—H1C	109.5	C12A—C13—C8	113.3 (3)
H1A—C1—H1C	109.5	C12A—C13—C12	29.1 (4)
H1B—C1—H1C	109.5	C8—C13—C12	112.79 (14)
C7—C2—C3	117.23 (18)	C12A—C13—H13A	84.1
C7—C2—C1	120.7 (2)	C8—C13—H13A	109.0
C3—C2—C1	122.1 (2)	C12—C13—H13A	110.7
C2—C3—C4	121.37 (18)	C12A—C13—H13B	129.0
C2—C3—H3A	119.3	C8—C13—H13B	109.1
C4—C3—H3A	119.3	C12—C13—H13B	107.4
C3—C4—C5	121.36 (18)	H13A—C13—H13B	107.8
C3—C4—H4A	119.3	C19—C14—C15	118.27 (17)
C5—C4—H4A	119.3	C19—C14—C12	116.22 (19)
C6—C5—C4	116.43 (16)	C15—C14—C12	125.5 (2)
C6—C5—C8	121.39 (15)	C19—C14—C12A	142.5 (4)
C4—C5—C8	122.14 (15)	C15—C14—C12A	98.9 (4)
C7—C6—C5	121.81 (18)	C12—C14—C12A	27.1 (3)
C7—C6—H6A	119.1	C16—C15—C14	121.29 (19)
C5—C6—H6A	119.1	C16—C15—H15A	119.4
C2—C7—C6	121.78 (19)	C14—C15—H15A	119.4
C2—C7—H7A	119.1	C15—C16—C17	120.66 (19)
C6—C7—H7A	119.1	C15—C16—H16A	119.7
C9—C8—C5	122.86 (15)	C17—C16—H16A	119.7
C9—C8—C13	119.62 (15)	C16—C17—C18	118.01 (17)
C5—C8—C13	117.49 (14)	C16—C17—C23	122.67 (18)
C8—C9—C10	123.74 (16)	C18—C17—C23	119.32 (17)
C8—C9—H9A	118.1	C19—C18—C17	119.70 (17)
C10—C9—H9A	118.1	C19—C18—C20	122.18 (18)
O1—C10—C9	122.38 (17)	C17—C18—C20	118.11 (18)
O1—C10—C11	120.41 (17)	C14—C19—C18	122.06 (18)
C9—C10—C11	117.01 (16)	C14—C19—H19A	119.0
O1—C10—C11A	118.7 (3)	C18—C19—H19A	119.0
C9—C10—C11A	110.6 (3)	C21—C20—C18	121.0 (2)
C11—C10—C11A	34.6 (3)	C21—C20—H20A	119.5
C25—C11—C12	109.87 (19)	C18—C20—H20A	119.5
C25—C11—C10	108.75 (17)	C20—C21—C22	120.7 (2)
C12—C11—C10	107.95 (17)	C20—C21—H21A	119.6
C25—C11—H11A	109.7	C22—C21—H21A	119.6
C12—C11—H11A	111.1	C23—C22—O4	125.6 (2)
C10—C11—H11A	109.5	C23—C22—C21	120.5 (2)
C25—C11—H12B	126.6	O4—C22—C21	113.9 (2)
C12—C11—H12B	72.7	C22—C23—C17	120.4 (2)
C10—C11—H12B	121.3	C22—C23—H23A	119.8
H11A—C11—H12B	38.4	C17—C23—H23A	119.8
C14—C12—C13	111.87 (15)	O4—C24—H24A	109.5
C14—C12—C11	112.17 (18)	O4—C24—H24B	109.5
C13—C12—C11	108.90 (19)	H24A—C24—H24B	109.5
C14—C12—H12A	107.8	O4—C24—H24C	109.5
C13—C12—H12A	107.6	H24A—C24—H24C	109.5
C11—C12—H12A	108.3	H24B—C24—H24C	109.5
C14—C12—H11B	118.0	O3—C25—O2	124.1 (2)
C13—C12—H11B	122.9	O3—C25—C11	128.3 (2)
C11—C12—H11B	76.6	O2—C25—C11	107.5 (2)
H12A—C12—H11B	31.9	O3—C25—C11A	93.5 (4)
C12A—C11A—C25	105.4 (7)	O2—C25—C11A	142.3 (4)
C12A—C11A—C10	99.5 (6)	C11—C25—C11A	34.8 (3)
C25—C11A—C10	101.5 (5)	C27—C26—O2	109.9 (3)
C12A—C11A—H12A	74.0	C27—C26—H26A	109.7
C25—C11A—H12A	132.8	O2—C26—H26A	109.7
C10—C11A—H12A	125.5	C27—C26—H26B	109.7
C12A—C11A—H11B	116.0	O2—C26—H26B	109.7
C25—C11A—H11B	116.1	H26A—C26—H26B	108.2
C10—C11A—H11B	116.1	C26—C27—H27A	109.5
H12A—C11A—H11B	41.9	C26—C27—H27B	109.5
C13—C12A—C11A	109.6 (7)	H27A—C27—H27B	109.5
C13—C12A—C14	104.7 (5)	C26—C27—H27C	109.5
C11A—C12A—C14	100.3 (7)	H27A—C27—H27C	109.5
C13—C12A—H11A	129.1	H27B—C27—H27C	109.5
C11A—C12A—H11A	79.0	C25—O2—C26	117.1 (2)
C14—C12A—H11A	123.5	C22—O4—C24	117.6 (2)
C13—C12A—H12B	113.7		
			
C7—C2—C3—C4	−0.7 (3)	C13—C12—C14—C15	84.7 (2)
C1—C2—C3—C4	179.2 (2)	C11—C12—C14—C15	−38.0 (3)
C2—C3—C4—C5	−0.4 (3)	C13—C12—C14—C12A	71.7 (7)
C3—C4—C5—C6	1.5 (3)	C11—C12—C14—C12A	−51.0 (6)
C3—C4—C5—C8	−176.30 (17)	C13—C12A—C14—C19	−50.3 (9)
C4—C5—C6—C7	−1.4 (3)	C11A—C12A—C14—C19	63.3 (8)
C8—C5—C6—C7	176.36 (19)	C13—C12A—C14—C15	123.0 (5)
C3—C2—C7—C6	0.8 (3)	C11A—C12A—C14—C15	−123.4 (6)
C1—C2—C7—C6	−179.1 (2)	C13—C12A—C14—C12	−67.6 (7)
C5—C6—C7—C2	0.3 (4)	C11A—C12A—C14—C12	46.0 (7)
C6—C5—C8—C9	169.74 (18)	C19—C14—C15—C16	−0.7 (3)
C4—C5—C8—C9	−12.6 (3)	C12—C14—C15—C16	177.92 (19)
C6—C5—C8—C13	−12.5 (2)	C12A—C14—C15—C16	−176.1 (3)
C4—C5—C8—C13	165.13 (16)	C14—C15—C16—C17	0.5 (3)
C5—C8—C9—C10	174.96 (17)	C15—C16—C17—C18	−0.1 (3)
C13—C8—C9—C10	−2.7 (3)	C15—C16—C17—C23	−179.20 (19)
C8—C9—C10—O1	−174.1 (2)	C16—C17—C18—C19	−0.1 (3)
C8—C9—C10—C11	11.0 (3)	C23—C17—C18—C19	179.05 (17)
C8—C9—C10—C11A	−26.3 (5)	C16—C17—C18—C20	−179.03 (18)
O1—C10—C11—C25	26.1 (3)	C23—C17—C18—C20	0.1 (3)
C9—C10—C11—C25	−158.9 (2)	C15—C14—C19—C18	0.5 (3)
C11A—C10—C11—C25	−71.3 (5)	C12—C14—C19—C18	−178.22 (16)
O1—C10—C11—C12	145.3 (2)	C12A—C14—C19—C18	173.1 (5)
C9—C10—C11—C12	−39.7 (3)	C17—C18—C19—C14	−0.2 (3)
C11A—C10—C11—C12	47.8 (5)	C20—C18—C19—C14	178.75 (17)
C25—C11—C12—C14	−57.1 (3)	C19—C18—C20—C21	−179.60 (18)
C10—C11—C12—C14	−175.51 (17)	C17—C18—C20—C21	−0.7 (3)
C25—C11—C12—C13	178.57 (17)	C18—C20—C21—C22	0.7 (3)
C10—C11—C12—C13	60.1 (2)	C20—C21—C22—C23	−0.2 (3)
O1—C10—C11A—C12A	−150.2 (5)	C20—C21—C22—O4	179.05 (19)
C9—C10—C11A—C12A	60.7 (7)	O4—C22—C23—C17	−179.54 (19)
C11—C10—C11A—C12A	−47.3 (6)	C21—C22—C23—C17	−0.3 (3)
O1—C10—C11A—C25	−42.2 (6)	C16—C17—C23—C22	179.5 (2)
C9—C10—C11A—C25	168.6 (3)	C18—C17—C23—C22	0.4 (3)
C11—C10—C11A—C25	60.6 (5)	C12—C11—C25—O3	−49.0 (3)
C25—C11A—C12A—C13	−176.4 (5)	C10—C11—C25—O3	68.9 (3)
C10—C11A—C12A—C13	−71.6 (8)	C12—C11—C25—O2	129.7 (2)
C25—C11A—C12A—C14	73.8 (7)	C10—C11—C25—O2	−112.3 (2)
C10—C11A—C12A—C14	178.6 (5)	C12—C11—C25—C11A	−46.5 (5)
C11A—C12A—C13—C8	47.6 (8)	C10—C11—C25—C11A	71.4 (5)
C14—C12A—C13—C8	154.4 (4)	C12A—C11A—C25—O3	−139.3 (6)
C11A—C12A—C13—C12	−47.7 (7)	C10—C11A—C25—O3	117.3 (4)
C14—C12A—C13—C12	59.1 (6)	C12A—C11A—C25—O2	36.8 (9)
C9—C8—C13—C12A	−6.8 (5)	C10—C11A—C25—O2	−66.5 (7)
C5—C8—C13—C12A	175.4 (5)	C12A—C11A—C25—C11	42.6 (6)
C9—C8—C13—C12	24.9 (2)	C10—C11A—C25—C11	−60.7 (5)
C5—C8—C13—C12	−152.93 (17)	O3—C25—O2—C26	2.4 (3)
C14—C12—C13—C12A	−81.3 (7)	C11—C25—O2—C26	−176.5 (2)
C11—C12—C13—C12A	43.3 (7)	C11A—C25—O2—C26	−173.0 (5)
C14—C12—C13—C8	−178.60 (17)	C27—C26—O2—C25	108.1 (3)
C11—C12—C13—C8	−54.1 (2)	C23—C22—O4—C24	−2.7 (4)
C13—C12—C14—C19	−96.6 (2)	C21—C22—O4—C24	178.0 (2)
C11—C12—C14—C19	140.7 (2)		

### Hydrogen-bond geometry (Å, °)

**Table:**

*D*—H···*A*	*D*—H	H···*A*	*D*···*A*	*D*—H···*A*
C26—H26A···O3^i^	0.97	2.42	3.332 (4)	157

Symmetry codes: (i) *x*, −*y*+3/2, *z*+1/2.
